# Supplementary figures and images for: Calotropis gigantea stem bark extract induced apoptosis related to ROS and ATP production in colon cancer cells
Source: PLoS One. 2021 Aug 3;16(8):e0254392. doi: 10.1371/journal.pone.0254392 (PMC8330925; doi:10.1371/journal.pone.0254392)

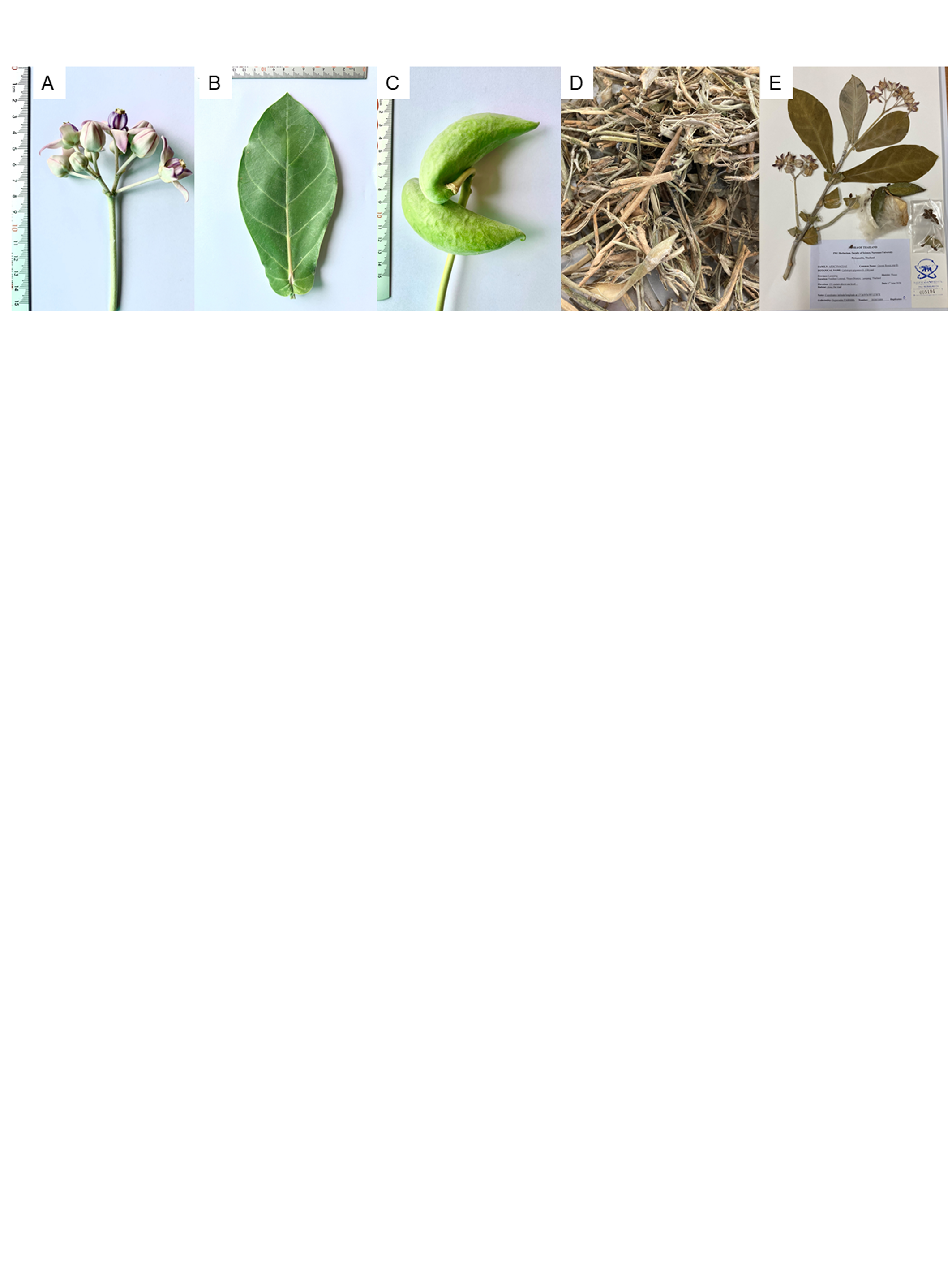

Supplement: S1 Fig — (A) flowers, (B) leaves, (C) fruits, (D) dry stem bark, and (E) the herbarium specimen. (TIF) [file pone.0254392.s001.tif]

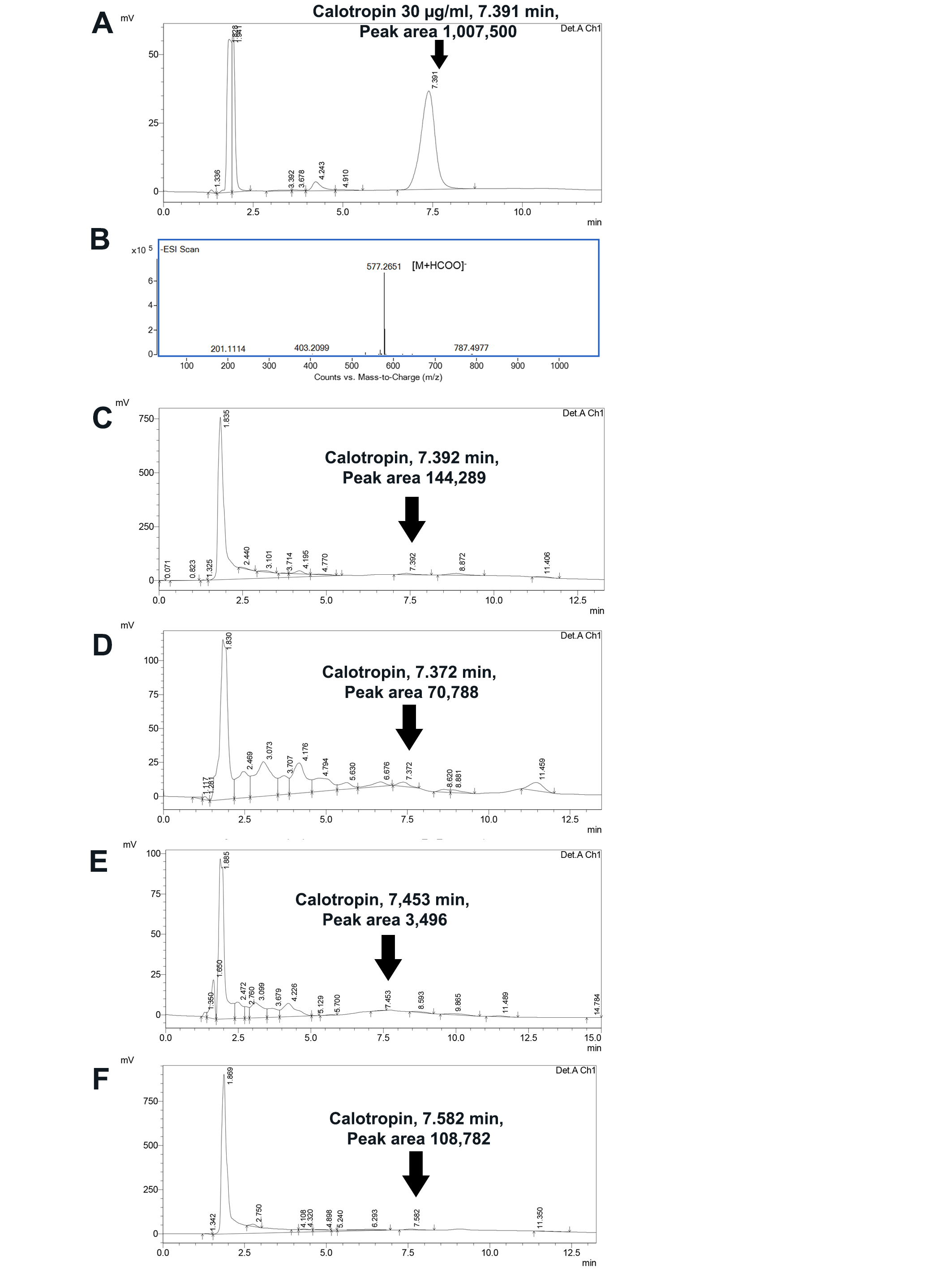

Supplement: S2 Fig — (A) Chromatogram of calotropin obtained upon HPLC analysis. (B) High resolution mass spectrum of calotropin. Chromatograms of CGEtOH (C), CGDCM (D), CGEtOAc (E), and CGW (F). Abbreviations: CGEtOH, C. gigantea ethanolic extract; CGDCM, C. gigantea dichloromethane extract; CGEtOAc, C. gigantea ethyl acetate extract; CGW, C. gigantea water extract. (TIF) [file pone.0254392.s002.tif]

Precision Plus Protein  
Dual Color Standards,  
500 µl #1610374

|               |   |      |   |   |    |      |      |      |
|---------------|---|------|---|---|----|------|------|------|
| CGDCM (µg/ml) | - | -    | 4 | 8 | 10 | 4    | 8    | 10   |
| 5-FU (µg/ml)  | - | 0.65 | - | - | -  | 0.65 | 0.65 | 0.65 |

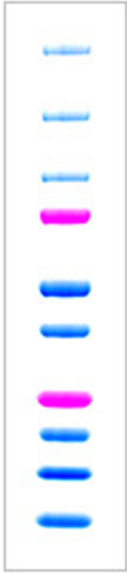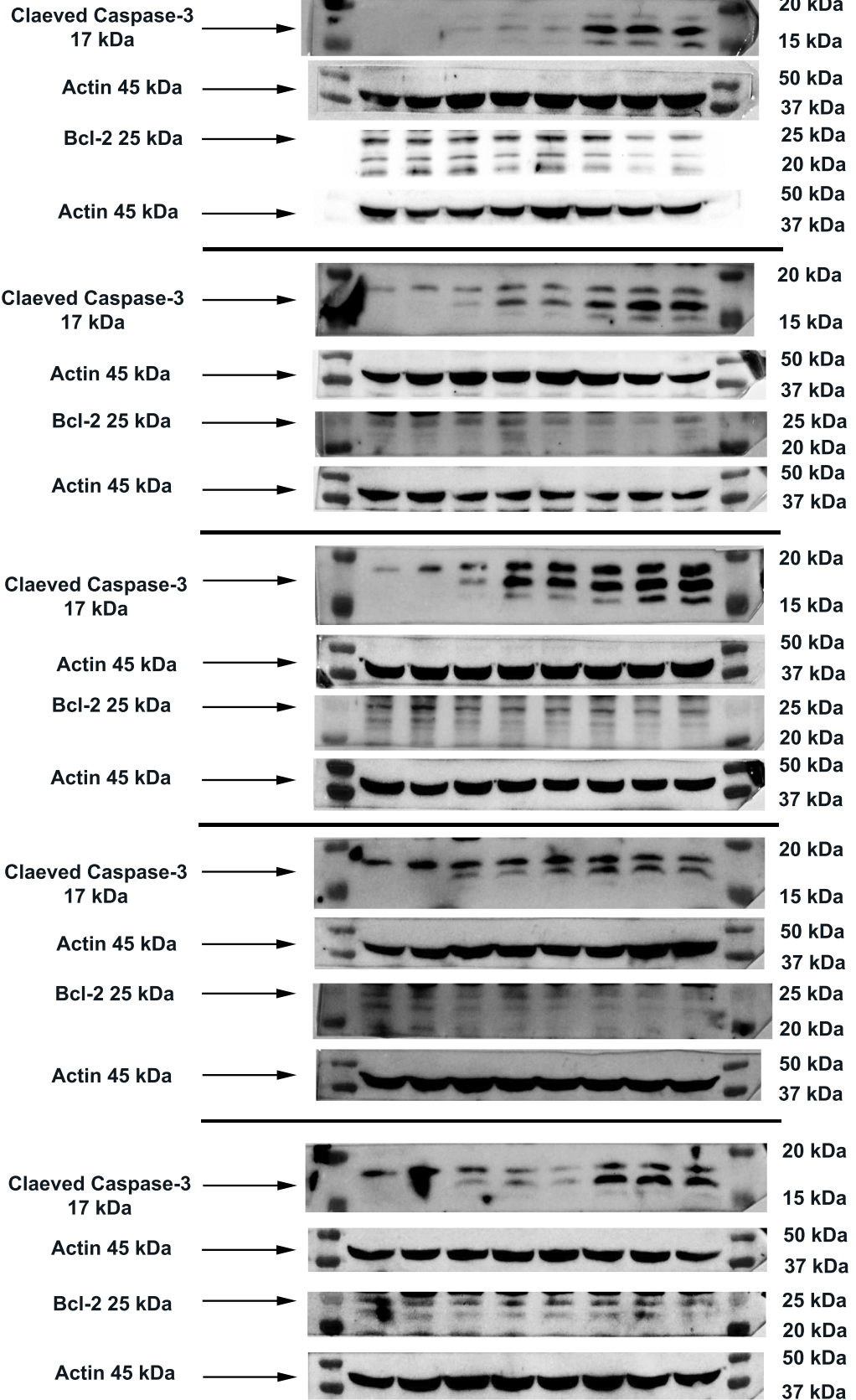

Supplement: S1 Raw images — (PDF) [file pone.0254392.s003.pdf]

Precision Plus Protein  
Dual Color Standards,  
500 µl #1610374

| CGDCM (µg/ml) | - | -    | 4 | 8 | 10 | 4    | 8    | 10   |
|---------------|---|------|---|---|----|------|------|------|
| 5-FU (µg/ml)  | - | 0.65 | - | - | -  | 0.65 | 0.65 | 0.65 |

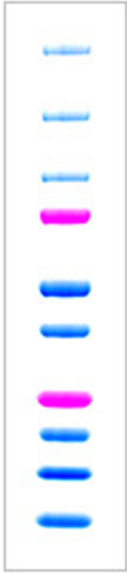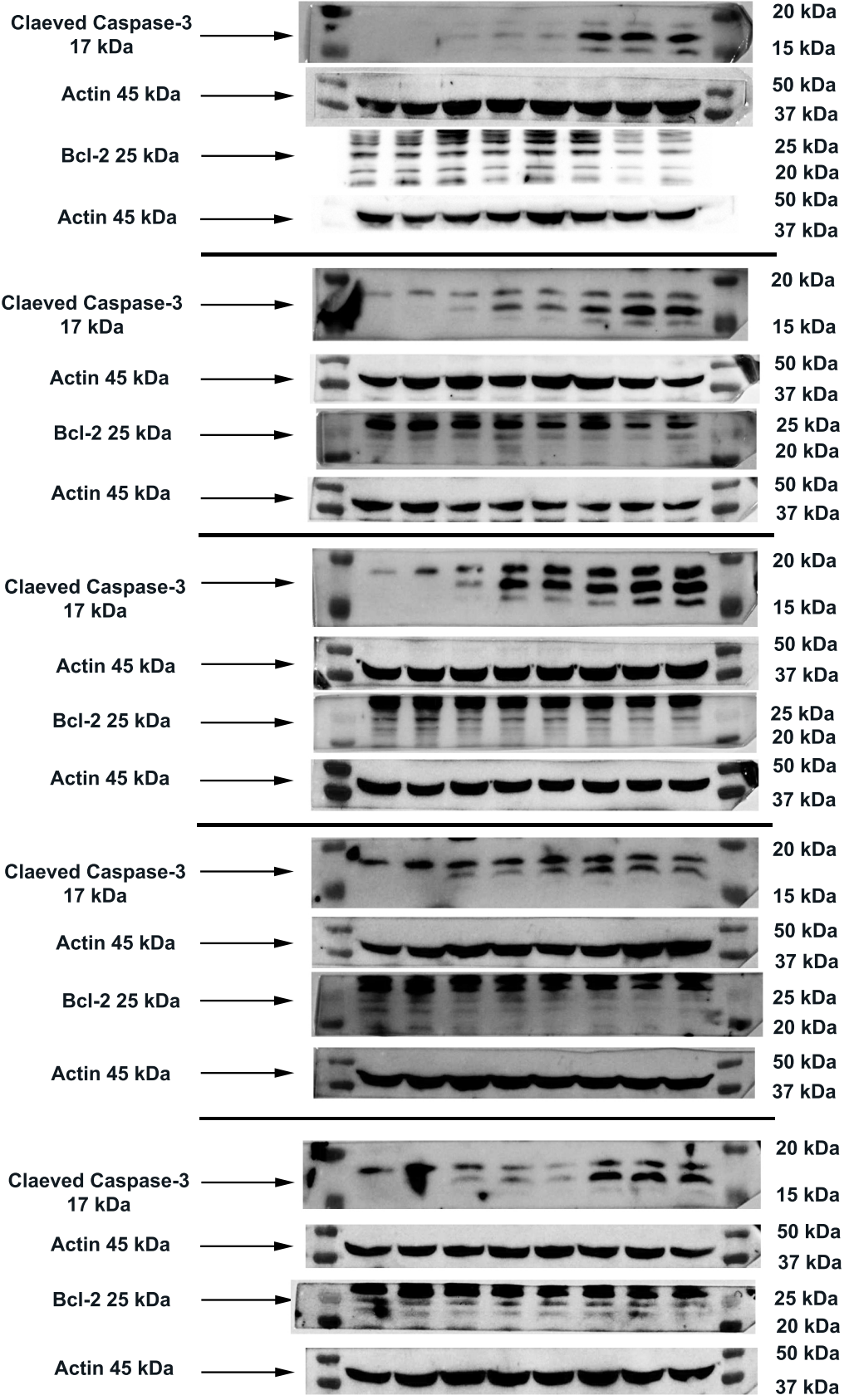

Supplement: S2 Raw images — (PDF) [file pone.0254392.s004.pdf]
